# Pulmonary Infection Caused by Mycolicibacterium neoaurum: The First Documented Case in Morocco

**DOI:** 10.7759/cureus.110189

**Published:** 2026-06-03

**Authors:** Abderrahim Oumloul, Sahar Rochd, Taoufik Ben Houmich, Asmae Lamrani Hanchi, Nabila Soraa

**Affiliations:** 1 Department of Microbiology, Faculty of Medicine and Pharmacy of Marrakesh, Cadi Ayyad University, Mohammed VI University Hospital, Marrakesh, MAR

**Keywords:** immunocompromised host, maldi-tof mass spectrometry, mycolicibacterium neoaurum, nontuberculous mycobacteria, pulmonary infection

## Abstract

Nontuberculous mycobacteria (NTM) are increasingly recognized as causes of human infection. *Mycolicibacterium neoaurum *(formerly *Mycobacterium neoaurum*)*,* a rapidly growing mycobacterium, is a rare pathogen, usually associated with catheter-related bloodstream infections. Pulmonary involvement is exceptional. We report the first documented case of pulmonary infection due to *Mycobacterium neoaurum* (*M. neoaurum)* in Morocco. A 39-year-old man with type 2 diabetes mellitus and refractory ulcerative colitis was hospitalized for severe autoimmune blistering disease and subsequently developed fever, productive cough, night sweats, and hypoxemia. Laboratory findings were indicative of an inflammatory syndrome. Chest imaging revealed a right upper lobe cavitary lesion. Bronchoalveolar lavage culture yielded acid-fast bacilli after eight days, identified as *M. neoaurum *by matrix-assisted laser desorption/ionization-time-of-flight mass spectrometry (MALDI-TOF). Empirical ciprofloxacin therapy administered for four weeks resulted in marked clinical and biological improvement. This case highlights the rare pulmonary pathogenicity of *M. neoaurum* and underscores the importance of considering uncommon NTM in cavitary lung disease, particularly in patients with complex comorbidities. Advanced diagnostic tools, such as MALDI-TOF, are crucial for accurate identification.

## Introduction

Nontuberculous mycobacteria (NTM) are environmental mycobacteria distinct from the *Mycobacterium tuberculosis* complex and are increasingly recognized as opportunistic pathogens capable of causing pulmonary, cutaneous, soft tissue, and disseminated infections [1,2]. Pulmonary NTM disease represents the most common clinical manifestation and may occur in both immunocompromised and immunocompetent individuals, particularly in patients with underlying structural lung disease or chronic comorbidities [2,3]. Clinically and radiologically, NTM pulmonary infections often mimic tuberculosis, bacterial lung abscesses, or malignancy, making diagnosis challenging and potentially delaying appropriate management [3,4]. The increasing incidence of NTM infections worldwide has been attributed to environmental changes, broader use of immunosuppressive therapies, aging populations, and improvements in microbiological diagnostic techniques [1,4].

*Mycolicibacterium neoaurum* (formerly *Mycobacterium neoaurum*) is a rapidly growing, pigmented mycobacterium belonging to the *M. parafortuitum* complex [5]. Human infections due to this organism remain rare and are most frequently associated with catheter-related bloodstream infections, particularly in immunocompromised patients [6,7]. Pulmonary involvement is exceptionally uncommon, with only a limited number of cases described in the literature [8]. Because of its rarity and nonspecific clinical presentation, recognition of *M. neoaurum* pulmonary infection may be difficult, highlighting the importance of advanced identification methods, such as matrix-assisted laser desorption/ionization-time-of-flight mass spectrometry (MALDI-TOF), mass spectrometry, and molecular techniques.

To our knowledge, this represents the first documented case of pulmonary infection due to *M. neoaurum* reported in Morocco.

## Case presentation

A 39-year-old male patient with type 2 diabetes mellitus receiving oral antidiabetic therapy, with ulcerative colitis (previously treated with azathioprine) and total colectomy, was admitted and managed at the Centre Hospitalier Universitaire Mohammed VI of Marrakesh, Morocco, for a rare association of dermatitis herpetiformis and superinfected bullous pemphigoid. During hospitalization, he developed a productive cough, night sweats, fever, and increased oxygen requirements.

Laboratory investigations revealed leukocytosis, mild anemia g/dL), and elevated C-reactive protein (CRP) (101 mg/L). Chest computed tomography (CT) scan demonstrated a thick-walled cavitary lesion in the right upper lobe, associated with adjacent nodular opacities and bronchiectatic changes (Figure 1).

**Figure 1:**
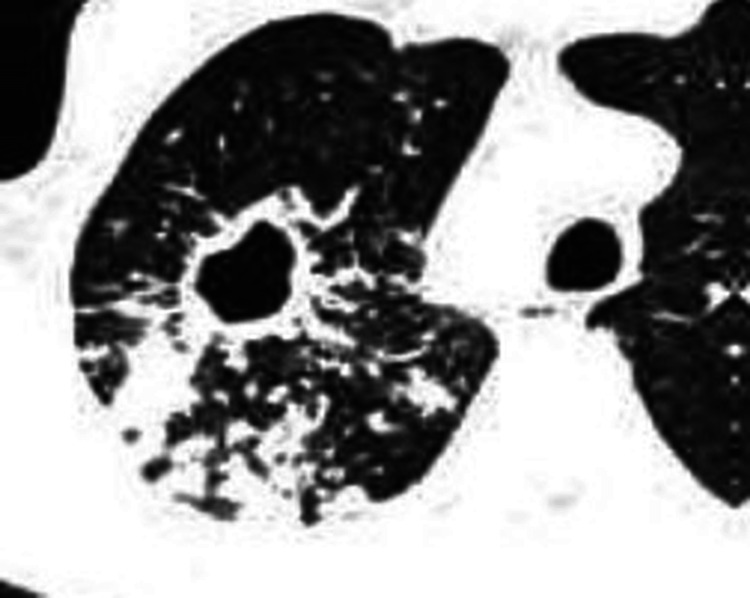
Chest CT showing a right upper lobe cavitary lesion Axial chest CT image demonstrating a thick-walled cavity in the right upper lobe with adjacent nodular opacities and bronchiectatic changes.

Bronchoscopy revealed no endobronchial abnormalities; however, acid-fast bacilli were isolated from bronchoalveolar lavage fluid after eight days of incubation on Löwenstein-Jensen medium, identified as *M. neoaurum* by MALDI-TOF, while direct smear microscopy remained negative (Figure 2). No other pathogens were isolated.

**Figure 2:**
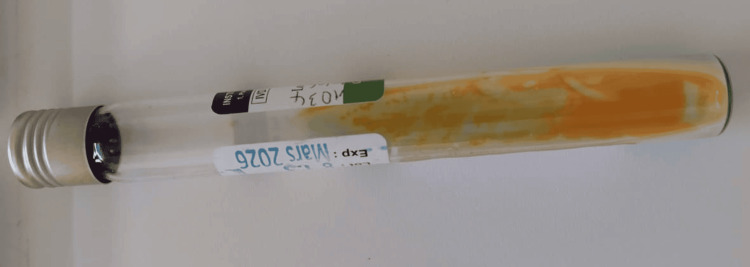
Culture of Mycobacterium neoaurum on a Löwenstein-Jensen medium

The patient received empirical oral ciprofloxacin at a dose of 500 mg twice daily for four weeks (susceptibility testing unavailable), resulting in clear clinical and biological improvement.

## Discussion

*Mycolicibacterium neoaurum* (formerly *Mycobacterium neoaurum*) is a rare cause of human infection, with pulmonary involvement remaining exceptional [6,9]. Most published cases describe catheter-related bloodstream infections or device-associated infections, which mainly occur in immunocompromised patients [9,10]. Against this background, the present case merits attention for two principal reasons: first, the pulmonary localization in the absence of any intravascular device at the time of diagnosis; and second, its status as, to our knowledge, the first documented case in Morocco, thus extending the known geographic distribution of this species [9,11].

The patient presented with multiple predisposing factors, including long-standing ulcerative colitis, prior immunomodulatory therapy, type 2 diabetes mellitus, chronic dermatologic disease, and prolonged hospitalization. These features are consistent with previously reported risk profiles for rapidly growing mycobacterial (RGM) infections, which frequently involve hosts with structural lung disease, malignancy, immunosuppression, or indwelling medical devices [1,6,10].

Although he was not neutropenic and no current immunosuppressive medication was being administered, his cumulative comorbidities and recent severe dermatologic condition likely contributed to a degree of immune dysfunction that facilitated NTM infection. This illustrates that *M. neoaurum* infection is not strictly confined to “classic” immunosuppression but may also occur in patients with complex chronic illnesses and repeated antibiotic exposure [3,10].

Pulmonary infections due to *M. neoaurum* have been described only in a handful of cases worldwide [8,9]. Clinical presentation is nonspecific and overlaps with other causes of cavitary lung disease, including tuberculosis, other NTM, bacterial abscess, and malignancy [1,12]. In the present case, the presence of the right upper lobe cavitary lesion in a country with a high prevalence of tuberculosis could easily have led to misdiagnosis as M. tuberculosis infection in the absence of species-level identification [12,13]. This underlines the importance of considering rare RGM in the differential diagnosis of smear-positive, culture-positive mycobacterial infections, particularly when the clinical course is atypical or when molecular testing for *M. tuberculosis* is negative [1,13].

Conventional phenotypic and biochemical methods often fail to reliably distinguish *M. neoaurum* from other rapidly growing mycobacteria [6,14]. In this context, the use of MALDI-TOF mass spectrometry played a central role in the present case by enabling rapid, species-level identification, in line with recent data emphasizing its utility for NTM and RGM identification in routine practice [14,15]. In settings where access to 16S rRNA sequencing or other molecular tools may be limited, MALDI-TOF represents a pragmatic, cost-effective alternative to improve recognition of rare species and avoid misdiagnosis [15,16].

There are currently no standardized treatment guidelines for *M. neoaurum *infections. Available data, derived from case reports and small series, suggest that isolates are frequently susceptible to fluoroquinolones, amikacin, macrolides, doxycycline, and certain β-lactams, such as cefoxitin [7,17]. For RGM infections in general, expert recommendations advocate the use of at least two active agents and prolonged therapy, especially for pulmonary disease [1,18]. In our case, ciprofloxacin monotherapy was initiated empirically and led to rapid clinical and biological improvement, which is consistent with the reported activity of fluoroquinolones against *M. neoaurum* [7,17].

Nevertheless, the treatment strategy in this case raises several important points. First, the absence of antimicrobial susceptibility testing is a significant limitation, as RGM may exhibit variable resistance patterns, and in vitro data would have strengthened therapeutic decisions [17,18]. Second, the relatively short duration of therapy (four weeks) contrasts with the prolonged regimens typically recommended for NTM pulmonary infections, which often extend for months to reduce the risk of relapse [1,18]. The favorable short-term outcome is encouraging, but long-term clinical and radiological follow-up is essential to exclude late recurrence or chronic colonization [1].

The present case also emphasizes the importance of a multidisciplinary approach in complex patients. The infection occurred in a dermatology ward, in a patient primarily admitted for severe autoimmune blistering disease. This context illustrates how NTM infections can emerge outside traditional high-risk units, such as oncology or intensive care [3,12]. Close collaboration between dermatologists, pulmonologists, microbiologists, and infectious disease specialists is therefore crucial for early recognition and appropriate management of atypical NTM presentations [1].

Several limitations must be acknowledged. As a single case report, our observations cannot be generalized to all patients with M. neoaurum pulmonary infection.

The lack of molecular resistance testing, imaging follow-up over an extended period, and data on potential environmental sources (water, hospital surfaces) limits our ability to conclude transmission or reservoirs [1,11]. However, the case remains highly informative in demonstrating the pathogenic potential of this species in a pulmonary context and the feasibility of diagnosing it with currently available tools in a middle-income country [14-16].

## Conclusions

This case adds to the scarce evidence implicating *M. neoaurum* in pulmonary infection. It underscores the need for heightened clinical suspicion of NTM, including rare species, in patients with cavitary pulmonary lesions and complex comorbidities. It also highlights the crucial role of advanced diagnostic methods, such as MALDI-TOF mass spectrometry, in accurately identifying unusual mycobacteria. Further case reports and series are needed to better characterize the clinical spectrum, antimicrobial susceptibility patterns, optimal therapeutic regimens, and long-term outcomes associated with *M. neoaurum* infections.
